# Prediction tool for early identification of patients at risk of Crohn’s disease in perianal fistulas and abscesses (PREFAB): Analysis of a prospective pilot study at a non-academic, teaching centre in the Netherlands

**DOI:** 10.1007/s10151-025-03209-0

**Published:** 2025-10-03

**Authors:** L. J. Munster, E. J. de Groof, S. van Dieren, M. W. Mundt, W. A. Bemelman, C. J. Buskens, J. D. W. van der Bilt

**Affiliations:** 1 Department of Surgery, Flevoziekenhuis, Almere, The Netherlands; 2 Department of Surgery, Amsterdam UMC (Location VUmc), De Boelelaan 1117, 1081 HV Amsterdam, The Netherlands; 3 Department of Gastroenterology and Hepatology, Flevoziekenhuis, Almere, The Netherlands

**Keywords:** Crohn’s disease, Perianal fistula, Faecal calprotectin, Early diagnosis

## Abstract

**Background:**

The aim of this study was to identify patients at risk of Crohn’s disease (CD) when presenting with perianal disease and to prospectively identify clinical characteristics (‘red flags’) associated with CD.

**Methods:**

All consecutive patients ≥ 16 years presenting with a perianal abscess (PAA)/fistula (PAF) between January and December 2022 were prospectively included. Faecal calprotectin (FCP) was measured in all patients, and patients were screened for potential red flags associated with CD by the use of a perianal red flags index (pRFI)-questionnaire. Colonoscopy was performed when FCP ≥ 150 mcg/g.

**Results:**

Overall, 115 patients were included (median age 38 years; IQR 28–53), 55 with PAA (48%) and 60 with PAF (52%). In total, 19 patients had FCP levels ≥ 150 mcg/g (median 381 mcg/g; IQR 191–1040), and were referred for colonoscopy, of which 10 were diagnosed with CD (9% of all patients; 17% of patients with PAF). Of all patients with PAF < 40 years, 29% were diagnosed with CD (9/31). During a minimal follow-up of 2 years, two colonoscopies were performed in patients with clinical suspicion for CD, demonstrating CD in 1 patient, resulting in a total of 11/115 patients with CD (10%), all presenting with PAF (18% of all patients with PAF). Univariate analysis showed that young age (< 40 years; odds ratio [OR] 4.9; 95% confidence interval [CI] 1.0–23.6), abdominal pains (OR 4.8; 95% CI 1.2–19.1), rectal bleeding (OR 4.3; 95% CI 1.2–15.6), fatigue (OR 3.9; 95% CI 1.1–14.4), multiple external (OR 6.0; 95% CI 1.5–24.6)/internal fistula openings (OR 61.2; 95% CI 9.8–383.4), fissures (OR 4.4; 95% CI 1.1–17.2), and proctitis (OR 22.9; 95% CI 1.9–277.5) increased the likelihood of having CD.

**Conclusion:**

With FCP-based screening for CD, approximately one in six patients with PAF, and even one in three patients with PAF < 40 years were diagnosed with CD. Therefore, FCP measurement is suggested in all patients with PAF, especially when they are < 40 years.

## Introduction

Perianal abscesses (PAA) and perianal fistulas (PAF) are life-impairing conditions that can be associated with Crohn’s disease (CD) [1, 2]. Diagnostic delay is common in patients with CD owing to the variability of its initial manifestations and its analogy with symptoms in cryptoglandular fistulas [3, 4], and this is associated with poor outcomes [5].

PAFs are the first manifesting sign in up to 10% of all patients with CD [3], but the recognition of a Crohn’s diagnosis in patients with idiopathic perianal disease has been given little attention up to now [6]. The median delay in diagnosis of CD in patients with perianal signs of CD is much longer when compared with patients presenting with abdominal complaints [7–9]. Patients with PAF who were eventually diagnosed with CD often underwent multiple prior perianal interventions before CD diagnosis was evident, which may result in damage to the anal sphincter complex and potentially more complex PAFs [10]. Complex PAFs are known to be associated with higher permanent -ostomy and proctectomy rates compared with simple PAFs [11]. In line with this, it was recently shown that a prolonged time to CD diagnosis in patients with PAFs is associated with poor long-term outcomes (e.g. higher defunctioning -ostomy and proctectomy rates) [5]. Therefore, early identification of underlying CD is crucial for potential curative treatment options [5, 11–14]. Recently, a perianal red flags index (pRFI) was developed, in which four red flags were identified for CD in patients presenting with a PAF (young age < 40 years, weight loss, abdominal pain and previous perianal interventions), and it was shown that a combination of these red flags yielded a good discriminative value of CD versus non-CD (area under the curve [AUC] 0.83; 95% confidence interval [CI] 0.72–0.94) [10]. As this index was based on a retrospective cohort study, where patients were asked to recall signs and symptoms encountered during their first PAF, it was impossible to include fistula characteristics. It might be hypothesized that this pRFI could be further improved by incorporating fistula characteristics and/or faecal calprotectin (FCP) measurement. Previously, it has been shown that a FCP of ≥ 150 mcg/g discriminates perianal CD from cryptoglandular PAF, even in the absence of intestinal ulcers [15]. For this reason, FCP might be a simple and non-invasive adjunct to differentiate patients with cryptoglandular perianal disease from those with CD. The aim of this study was to identify patients at risk of having CD presenting with perianal disease by the use of FCP and to prospectively identify red flags associated with CD.

## Methods

### Inclusion and exclusion criteria

In this single-centre prospective pilot study, all consecutive patients ≥ 16 years presenting with a PAA or PAF (primary or recurrent) at the outpatient clinic or emergency department were included during a period of 1 year (from 1 January 2022 to 31 December 2022) at a non-academic teaching hospital in the Netherlands. Patients were excluded if they were already diagnosed with CD or if CD was recently (less than 12 months ago) ruled out (by ileocolonoscopy, magnetic resonance enterography [MRE] or video capsule endoscopy [VCE]). Additionally, patients with another well-known aetiology for their perianal disease (e.g. pilonidal sinus, iatrogenic, malignancy, hidradenitis, sexually transmitted diseases and tuberculosis or obstetric-related [rectovaginal] fistulas) were excluded. Also, patients undergoing immunotherapy or chemotherapy, patients in whom the attending physician did not consider further diagnostics for CD to be useful (e.g. a poor prognosis due to another medical condition), patients declining to participate in answering the questionnaire or stools and patients lost to follow-up were excluded.

### Data collection

FCP was measured in all patients. In addition, patients were screened for red flags associated with CD by the use of a 24-item perianal red flags index questionnaire including characteristics potentially suggestive of CD and fistula characteristics as derived from literature, supplemented with items from the International Organization for the Study of Inflammatory Bowel Diseases (IO-IBD) red flags index for luminal disease [10, 16]. Baseline characteristics (e.g. age and gender) were collected in all patients. The number of ileocolonoscopies (including the percentage of positive ileocolonoscopies), small bowel assessments by magnetic resonance elastography (MRE) and/or video capsule endoscopy (VCE, including the percentage of positive MRE/VCEs) were collected. The median time from first PAA/PAF to CD diagnosis was assessed. All patients who were eventually not diagnosed with CD were called after a minimal follow-up of 2 years, and patient records were screened to check whether these patients developed any signs of CD over time.

### Outcomes

The primary outcome of this study was the amount of patients diagnosed with CD when presenting with a PAA or PAF using the use of FCP. Secondary outcomes were the clinical red flags (including fistula characteristics) associated with CD and the median time from first PAA/PAF to CD diagnosis.

### Faecal calprotectin measurement

Since 2017, FCP measurement has been performed as part of regular diagnostic workup by use of a quantitative, in vitro diagnostic immunoassay, the DiaSorin LIAISON calprotectin assay (DiaSorin S.p.A, Crescentino SNC, 13,040 Saluggia [VC] – Italy) in our hospital. Measurement was performed according to the manufactures’ instructions with processing of all stool samples within a maximum of 3 days following collection. The upper limit of detection in this test was 8000 mcg/g, and to conduct uniform results, no other measurement tools were used. Elevated FCP levels were defined as ≥ 150 mcg/g [15]. Due to potentially elevated FCP levels at the time of an active PAA, FCP measurement was repeated 6 weeks after the first presentation in patients presenting with a PAA in whom FCP levels initially were elevated.

### Criteria for referral and diagnostic workup

Patients were referred for further diagnostic workup (ileocolonoscopy, conforming with European Crohn’s and Colitis Organisation [ECCO] guidelines) for CD when FCP levels ≥ 150 mcg/g [15, 16]. All ileocolonoscopies were performed by a dedicated Inflammatory Bowel Disease (IBD) gastroenterologist and included random biopsies of the ileum and colon. In the case of a negative ileocolonoscopy but persistent suspicion of CD, VCE examination (or MRE examination in the case of suspected bowel obstruction) was performed.

### Ethics

Since this study was part of the development of a new local healthcare protocol, this study did not fall within the scope of the Medical Research Involving Human Subjects Act (WMO, decided by the accredited medical ethics review committee [METC]; reference number W21_531#21.587). However, this project was conducted according to the principles of the Declaration of Helsinki (64th WMA General Assembly, Fortaleza, Brazil, October 2013) and according to the General Data Protection Regulation (GDPR).

### Statistical analyses

Continuous data were presented as mean (standard deviation [SD]) or median (interquartile range [IQR]) depending on distribution. Categorical data were presented as number of patients including proportions. Univariate analyses were performed on all (fistula) characteristics to identify potential red flags associated with CD diagnosis. A two-tailed *p*-value of less than 0.05 was considered significant. All data were collected and analysed in Statistical Package for the Social Sciences (SPSS) Statistics (Windows, version 22, IBM Crop., Armonk, New York, USA).

## Results

**Fig. 1:**
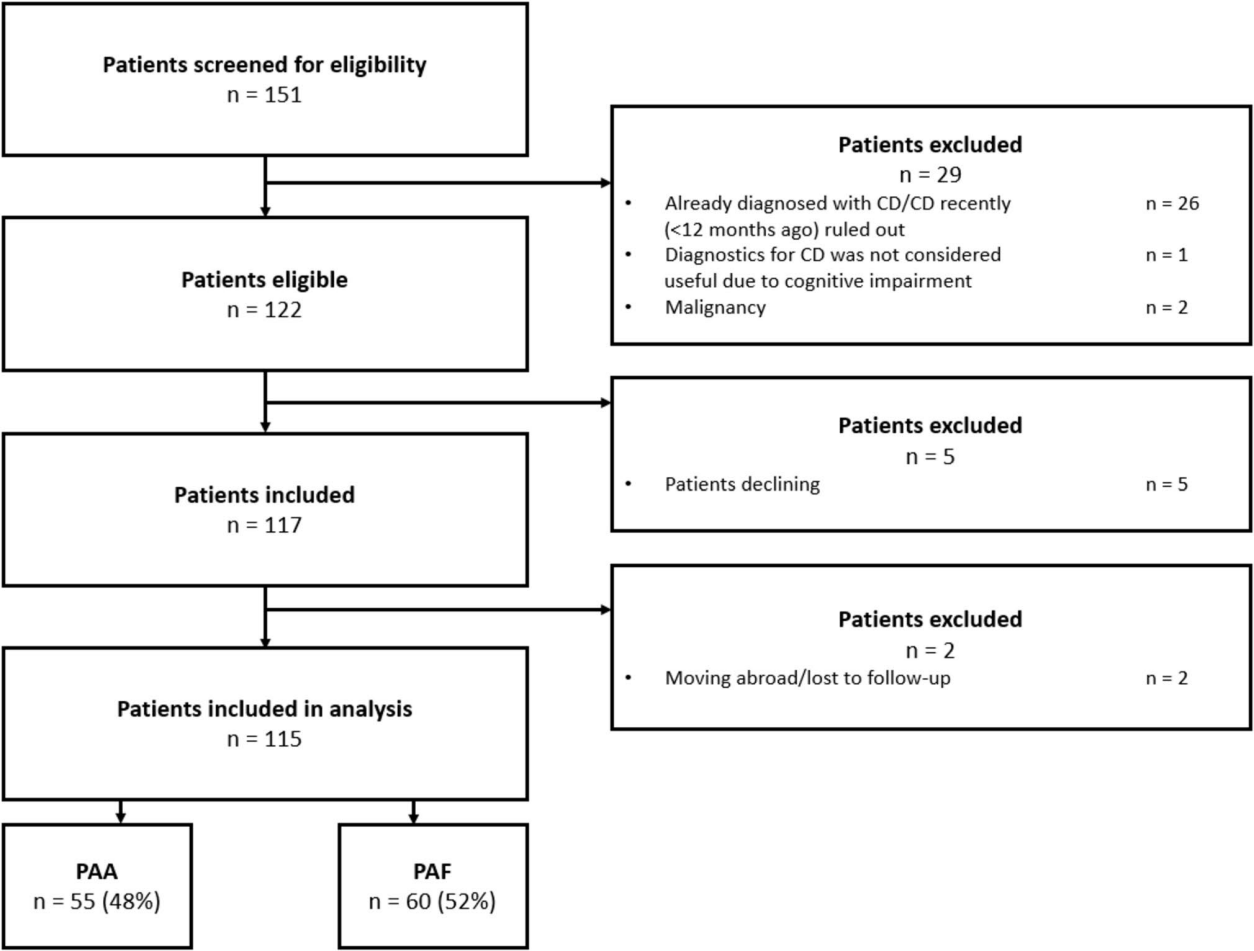
Patient flowchart

A total of 151 patients were screened for eligibility (see Fig. 1 for a patient flowchart), of which 115 patients were included in the analysis (73% men), with a median age at presentation of 38 (IQR 28–53) years. In all, 55 patients (48%) presented with a PAA (and no signs of fistula formation), and 60 patients (52%) presented with a PAF (Table 1). A total of 55 patients (48%) presented with a recurrent PAA or PAF. All patients underwent surgical drainage or seton placement in the emergency department (ED) or in theatre. In all, 19 patients presented with FCP levels ≥ 150 mcg/g (median 381 mcg/g; IQR 191–1040) and were referred for colonoscopy, of which 10 were diagnosed with CD (9% of all patients).

**Table 1:** Baseline characteristics of all included patients combined as well as divided into a PAA and PAF group at presentation

Characteristics	Total (PAA/PAF)	PAA	PAF
*n* (%)	115	55 (48)	60 (52)
Men, *n* (%)	84 (73)	39 (71)	45 (75)
Age, median (IQR)	38 (28–53)	39 (31–53)	38 (28–53)
Age < 40 years, *n* (%)	59 (51)	28 (51)	31 (52)
FCP > 150 mcg/g, *n* (%)	19 (17)	2 (4)	17 (28)
FCP (mcg/g), median (IQR)	32 (12–109)	17 (10–55)	68 (12–175)
Colonoscopy, *n* (%)	21 (18)	3 (6)	18 (30)

*IQR* interquartile range; *pAA* perianal abscess; *pAF* perianal fistula, *FCP* faecal calprotectin

After a minimal follow-up of 2 years, none of the patients with a healing PAA were diagnosed with CD. CD was demonstrated in one patient originally presenting with a PAF after 13 months, meaning that all patients eventually diagnosed with CD originally presented with a PAF (10% of all patients, and 18% of all patients presenting with PAF, of whom 17% were identified by active screening using FCP; Table 2). The median time from onset of the PAF to CD diagnosis was 39 months (IQR 11–56). In patients presenting with a primary PAF (e.g. when excluding patients with a recurrent PAF), the median time from onset of the PAF to CD diagnosis was 3 months (IQR 0–5). Median FCP levels of patients with CD diagnosis were 552 mcg/g (IQR 271–1180) as compared with 24 mcg/g (IQR 10–90) in patients with cryptoglandular disease (*p* < 0.001).

**Table 2:** Diagnostic outcomes of all included patients

	Total (PAA/PAF) *n* = 115
CD diagnosis, *n* (%)	11 (10)
CD diagnosis in PAF (total *n* = 60), *n* (%)	11 (18)
Age at diagnosis, *n* (%)	
Montreal A1	0 (0)
Montreal A2	9 (82)
Montreal A3	2 (18)
Location of disease, *n* (%)^*^	
Montreal L1	6 (55)
Montreal L2	2 (18)
Montreal L3	1 (9)
Behaviour of disease at diagnosis, *n* (%)	
Montreal B1	9 (82)
Montreal B2	1 (14)
Montreal B3	1 (14)
Time from first PAF to CD diagnosis (months), median (IQR)	39 (11–56)
Time from first PAF to CD diagnosis in patients with a primary PAF (e.g. recurrences excluded) (months), median (IQR)	3 (0–5)

*IQR* interquartile range; *pAA* perianal abscess; *pAF* perianal fistula

^*^In two patients, CD was based on clinical characteristics (> 10 external fistula openings and the suspicion of cutaneous CD)

Of patients with a negative ileocolonoscopy (*n* = 14), six underwent additional small bowel examination due to persistent clinical suspicion by either VCE (*n* = 4, all negative) or MRE (*n* = 2, both negative). A total of nine patients were endoscopically or histologically diagnosed with CD. In total, CD was based on clinical characteristics (> 10 external fistula openings and the suspicion of cutaneous Crohn’s) in two patients.

### Red flags associated with CD in patients with PAF

The number of patients with cryptoglandular disease and patients with CD with positive red flags from the pRFI are presented in Table 3 (clinical characteristics) and Table 4 (fistula characteristics/characteristics on physical examination). The median age of patients with Crohn’s who had PAF was 27 (IQR 24–30) years as compared with 41 (IQR 31–54) years in patients presenting with cryptoglandular disease, with 29% of all patients with PAF < 40 years being diagnosed with CD (9/31). Univariate analysis showed that young age < 40 years (odds ratio [OR] 4.9; 95% CI 1.0–23.6; *p* < 0.001), abdominal pains (OR 4.8; 95% CI 1.2–19.1; *p* = 0.036), rectal bleeding (OR 4.3; 95% CI 1.2–15.6; *p* = 0.034), fatigue (OR 3.9; 95% CI 1.1–14.4; *p* = 0.043), multiple external fistula openings (OR 6.0; 95% CI 1.5–24.6; *p* = 0.021), multiple internal fistula openings (OR 61.2; 95% CI 9.8–383.4; *p* < 0.001), fissures (OR 4.4; 95% CI 1.1–17.2; *p* = 0.046) and proctitis (OR 22.9; 95% CI 1.9–277.5; *p* = 0.024) increased the likelihood of having CD.

**Table 3:** Univariate analysis of all potential clinical red flags in patients with CD compared with patients without CD

Red flags	Cryptoglandular disease *n* = 104	CD *n* = 11	OR (95% CI)	*p*-Value
Age, median (IQR)	41 (31–54)	27 (24–30)	NA	0.003^*^
Age < 40 years, *n* (%)	50 (48)	9 (82)	4.9 (1.0–23.6)	< 0.001^*^
Gender, men (%)	77 (74)	7 (64)	0.6 (0.2–2.3)	0.484
BMI, median (IQR)	27 (24–30)	26 (25–32)	NA	0.668
Smoking, *n* (%)	45 (43)	2 (18)	0.3 (0.1–1.4)	0.195
Family history, *n* (%)	8 (8)	1 (9)	1.2 (0.1–10.6)	1.000
Weight loss, *n* (%)	10 (10)	2 (18)	2.1 (0.4–11.0)	0.322
Abdominal pains, *n* (%)	11 (11)	4 (36)	4.8 (1.2–19.1)	0.036^*^
Abdominal pain childhood, *n* (%)	13 (13)	4 (36)	4.0 (1.0–15.6)	0.057
Diarrhoea, *n* (%)	10 (10)	2 (18)	2.1 (0.4–11.0)	0.322
Rectal bleeding, *n* (%)	17 (16)	5 (46)	4.3 (1.2–15.6)	0.034^*^
Fatigue, *n* (%)	32 (31)	7 (64)	3.9 (1.1–14.4)	0.043^*^
Anaemia, *n* (%)	7 (7)	2 (18)	3.1 (0.6–17.1)	0.206
Vitamin deficiencies, *n* (%)	20 (19)	5 (46)	3.5 (0.9–12.6)	0.059
Gastrointestinal complaints, *n* (%)	12 (12)	2 (18)	1.7 (0.3–8.8)	0.622
Bowel surgery in the past, *n* (%)	8 (8)	1 (9)	1.2 (0.1–10.6)	1.000
Other perianal complaints, *n* (%)	28 (27)	3 (27)	1.0 (0.3–4.1)	1.000
Extraintestinal complaints, *n* (%)	23 (22)	1 (9)	0.4 (0.0–2.9)	0.454
Number of previous perianal interventions, median (IQR)	0 (0–2)	1 (1–3)	NA	0.234

*CD* Crohn’s disease, *IQR* interquartile range, *CI* confidence interval, *NA* not applicable

^*^Significant *p*-value

**Table 4:** Univariate analysis of all characteristics on physical examination in patients with CD compared with patients without CD

Red flags	Cryptoglandular disease *n* = 104	CD *n* = 11	OR (95% CI)	*p*-Value
Multiple external fistula openings, *n* (%)	9 (9)	4 (36)	6.0 (1.5–24.6)	0.021^*^
Multiple internal fistula openings, *n* (%)	2 (2)	6 (55)	61.2 (9.8–383.4)	< 0.001^*^
Anterior fistula opening, *n* (%)	18 (17)	4 (36)	2.7 (0.7–10.3)	0.217
Fissures, *n* (%)	12 (12)	4 (36)	4.4 (1.1–17.2)	0.046^*^
Proctitis, *n* (%)	1 (1)	2 (18)	22.9 (1.9–277.5)	0.024^*^

*CD* Crohn’s disease, *IQR* interquartile range, *CI* confidence interval, *NA* not applicable

^*^Significant *p*-value

## Discussion

In this pilot study, using standardized FCP measurement in all consecutive patients presenting with a PAA or PAF, we have been able to diagnose CD in one out of six patients presenting with PAF. In this series, no CD was demonstrated in patients presenting with PAA without a fistula. In patients < 40 years presenting with a PAF, we found CD in 29%, which is approximately one-third of all patients < 40 years presenting with a PAF. In addition to young age (< 40 years) and the presence of a PAF in general, several potential red flags for CD in patients with PAF were identified, including abdominal pains, rectal bleeding, fatigue, multiple external fistula openings, multiple internal fistula openings, fissures and proctitis, which all significantly increased the likelihood of having CD. However, although patients in this study were not referred to further diagnostics on the basis of red flags (but only on elevated FCP levels), the results of this study suggest that the added value of most red flags on top of structural FCP measurement appears to be minimal, given that most patients with CD were identified by the use of FCP measurement solely. However, the results of this study also emphasize that FCP measurement is relevant in specific patients with red flags such as younger age or with multiple internal/external fistula openings. Therefore, on the basis of this study, it is suggested to measure FCP levels in all patients presenting with a PAF, especially when they are < 40 years of age or when they present with multiple internal/external fistula openings.

Recently, the International Organization for Inflammatory Bowel Disease (IO-IBD) developed and validated an index tool to identify early CD and reduce diagnostic delay, showing that the index combined with faecal calprotectin (FCP) measurement was a valid tool to identify patients with high probability of having CD at an early stage when presenting with abdominal complaints [16]. However, this tool was not validated in patients presenting with PAF, which is remarkable, as a PAF is the manifesting sign in around 10% of all patients with CD. [3]

As mentioned, identifying alarming symptoms in patients presenting with perianal disease can be challenging owing to the variability and subtlety of its initial manifestations [3–5]. Moreover, inexperience of general, non-IBD surgeons may contribute to the failure of recognizing Crohn’s PAF, which could result in a significant delay in diagnosis. Reported delay in diagnosis of CD in those patients specifically in the literature varies widely, but diagnosis may take up to several years, with a mean time to CD diagnosis of almost 4 years in literature [3]; this is in line with the results of this study (median time from first PAF to CD diagnosis 39 months; IQR 11–56). It is assumed that this prolonged time to diagnosis was caused by the inclusion of patients with primary as well as recurrent PAFs (e.g. patients referred from other centres and those with long-term complaints) since the median time to CD diagnosis was significantly lower when interpreting the results of patients with a primary PAF solely (e.g. when excluding recurrences, the median time from first PAF to CD diagnosis was 3 months; IQR 0–5). It was recently showed that a prolonged time to CD diagnosis in patients with PAFs is associated with poor long-term outcomes [5]. In line with this, it was shown that patients with radiological healing, nowadays the best predictor for no recurrence [17], had the shortest time to CD diagnosis (median 4 months; IQR 2.0–16.5), as compared with patients in whom only clinical closure could be achieved [5]. Therefore, it is of the utmost importance that patients presenting with a PAF and at risk of having CD will be identified at an early stage, and that clinical awareness of underlying CD in these patients will increase. It is assumed that the use of a simple and practical prediction tool, including FCP measurement in patients with PAF, at an early stage could potentially contribute to a reduced delay in diagnosis; this is especially relevant in patients with red flags such as younger age or with multiple internal/external fistula openings. Since results of CD fistula treatment are still disappointing, even with the best available therapies [17], early diagnosis may improve outcomes by initiating early multidisciplinary IBD-directed treatment, thereby preventing complex fistula formation, recurrent surgeries and potentially -ostomy and/or proctectomies.^5,11^

### Strengths and limitations

The main limitation of this study is the relatively small sample size precluding multivariate analysis of predictive factors. However, this is one of the largest consecutive prospective studies identifying risk factors for CD in patients presenting with perianal CD, with only 6% (7/122) of all eligible patients not included in the analysis, resulting in a high external validity. Another limitation is the lack of the gold standard (ileocolonoscopy) in all patients. However, this series reflects daily clinical practice and shows that, with a simple tool, CD can be diagnosed in a large proportion of patients with PAF. The follow-up of a minimal 24 months with only one ‘missed CD diagnosis’ with active screening also suggests that FCP testing in patients presenting with a PAF is justified to reduce delay in CD diagnosis, thereby hopefully improving the natural history of CD and its associated PAF. However, it should be kept in mind that the follow-up of ‘only’ 2 years could potentially have resulted in an underestimation of the real amount of patients with CD since the median time to CD diagnosis in this study was more than 3 years.

Since FCP testing is relatively low cost and easy to use in daily clinical practice, it can easily be used as an adjunct for raising suspicion of CD in patients with PAF. Moreover, potential high healthcare costs due to negative outcomes in patients with a prolonged time to CD diagnosis (e.g. a defunctioning -ostomy or proctectomy) could be reduced. However, it should be kept in mind that unnecessary ileocolonoscopies should be avoided at all times, and it would be best to identify patients with PAF at the highest risk of having CD. In this study, it was attempted to identify additional red flags suggestive of CD in patients presenting with perianal disease by conducting structured tracking of patients’ complaints. Although it was assumed that these clinical signs/symptoms could be used in daily practice to increase awareness and reduce delay in CD diagnosis, the results of this study suggest that the added value of most red flags on top of structural FCP measurement appears to be minimal. However, due to the relatively small sample size in this study, the added value of any potential red flags in daily clinical practice could be underestimated. In line with this it was not possible to reliably identify the optimal number needed to screen to find one patient with perianal CD. Therefore, the results of this study should be validated in larger, prospective multicentre studies, and longer follow-up periods need to be achieved to identify which patient-specific red flags are suitable for implementation in a new, clinical decision tool which could be used in daily clinical practice so that only patients at high risk of having CD will be subject to further diagnostics.

## Conclusion

With active screening for CD based on FCP measurement with cut-off ≥ 150, approximately one out of six patients – and even one out of three patients < 40 years – presenting with a PAF were diagnosed with CD. On the basis of this study, it is recommended to measure FCP levels in all patients presenting with a PAF, especially when they are < 40 years of age or when they present with multiple internal/external fistula openings. The added value of potential red flags for CD in patients with PAF should be further explored. Earlier diagnosis of CD might improve the natural history of CD and its associated PAFs, but longer follow-up and validation studies will follow.

### What is already known

A prolonged time to CD diagnosis in patients with a PAF as a manifesting sign is common, and is associated with worse long-term outcomes.

### What is new here

In patients presenting with perineal disease, no underlying CD was found in patients with an abscess. However, by screening for CD in patients presenting with a PAF, using standardized FCP measurement, CD was diagnosed in one out of six patients – and even in one out of three patients <40 years – presenting with a PAF. FCP measurement should be used in daily practice to reduce delay in CD diagnosis, thereby hopefully improving the natural history of CD and its associated PAF.

### How can this study help patient care

This series reflects daily clinical practice and shows that, with a simple tool, CD can be diagnosed in a large proportion of patients with PAF. By implementing standard FCP as a screening tool and increasing awareness for CD in patients with PAF in general, the time to CD diagnosis might be reduced. This can contribute to better long-term outcomes, including potentially higher radiological fistula healing rates, lower -ostomy and/or proctectomy rates and reduced healthcare costs.

## Data Availability

The authors of the manuscript confirm that all data supporting the findings of this pilot study are available within the manuscript. Supplementary details are available on reasonable request.
